# Moxibustion Reduces Inflammatory Response in the Hippocampus of a Chronic Exercise-Induced Fatigue Rat

**DOI:** 10.3389/fnint.2019.00048

**Published:** 2019-09-20

**Authors:** Tian-ge Li, Ling Shui, Dong-yu Ge, Rong Pu, Shu-mei Bai, Jun Lu, Ying-song Chen

**Affiliations:** ^1^School of Acupuncture-Moxibustion and Tui Na, Beijing University of Chinese Medicine, Beijing, China; ^2^School of Inner Mongolia Medicine, Inner Mongolia Medical University, Huhehaote, China; ^3^School of Traditional Chinese Medicine, Beijing University of Chinese Medicine, Beijing, China; ^4^The Eighth Medical Center of PLA General Hospital, Beijing, China

**Keywords:** exercise-induced fatigue, exhaustive swimming, inflammation, moxibustion, pro-inflammatory cytokines

## Abstract

Accumulating data indicates that brain inflammation plays an important role in the pathophysiology of chronic exercise-induced fatigue. Moxibustion in traditional Chinese medicine has been found to alleviate exercise-induced fatigue. However, it remains unclear whether the effect of moxibustion is related to its anti-inflammatory properties. In this study, rats were exposed to 3-week exhaustive swimming to induce chronic exercise-induced fatigue. The body weight, exhaustive swimming time, tail suspension test and open-field test were observed. Real-time polymerase chain reaction (RT-PCR) was used to determine the mRNA expression of proinflammatory cytokines (interleukin-1β [IL-1β], interleukin-6 [IL-6], and tumor necrosis factor-α[TNF-α]), and enzyme-linked immunosorbent assay (ELISA) was used to detect IL-1β, IL-6, and TNF-α concentrations. Chronic exhaustive exercise significantly reduced the body weight and exhaustive swimming time, and increased tail suspension immobility time, which were reversed by moxibustion treatment. Compared with control rats, the mRNA and protein expression of IL-1β, IL-6, and TNF-α in the hippocampus was significantly increased in exhaustive swimming trained rats. Moxibustion significantly decreased the level of IL-6 in the hippocampus, but not affected IL-1β and TNF-α level significantly. Our results suggested that a potential inflammatory damage in the brain may be involved during chronic exhaustive exercise-induced fatigue. Moxibustion could attenuate the inflammatory impairment in exercise-induced fatigue, which might be mediated by inhibition of the proinflammatory cytokine IL-6 levels in the brain region.

## Introduction

Exercise-induced fatigue, which is a reduction in maximal voluntary muscle force that results from intense and prolonged exercise (Gandevia, 2001), may lead to a drop in physical performance. Proinflammatory cytokines in the brain play crucial roles in modulating the inflammatory response to exercise and have been regarded as contributing to exercise-induced fatigue (Vargas and Marino, 2014). Exercise can have favorable and harmful effects on the body depending on its intensity and duration. Regular and moderate exercise can have beneficial effects on immunity (Weinhold et al., 2016; Dantas et al., 2019), as well as improving LPS-induced learning and memory impairments by attenuating the hippocampal cytokine levels (Jahangiri et al., 2019). However, exhaustive exercise has been shown to reduce immune function, increase the risk of infection, and contribute to fatigue (Banfi et al., 2008; Kakanis et al., 2010; Shaw et al., 2018; Castell et al., 2019). Prolonged or strenuous exercise can induce a remarkable increase in circulating and cerebral proinflammatory cytokines such as interleukin-1β (IL-1β), interleukin-6 (IL-6) and tumor necrosis factor-α (TNF-α) (Fehrenbach and Schneider, 2006; Liu et al., 2017). For example, clinical study has found that the overexpression of proinflammatory cytokines have been observed in the blood after exhaustive exercise (Suzuki et al., 2002). It has been reported that exhaustive treadmill running in rats increased IL-1β and TNF-α level in the hippocampus (Sun et al., 2014). Another study indicated that eccentric exercise significantly increased the concentration of IL-1β in rat brain regions, and its levels correlated significantly with post-exercise delayed recovery and decreased exercise performance (Carmichael et al., 2005; Morris et al., 2015).

Moxibustion is a traditional Chinese medicine that uses the heat generated by burning Chinese mugwort, to stimulate acupuncture points of the body. Moxibustion has been used in China to treat diseases for more than 2000 years. It has been used to alleviate fatigue-related diseases, such as exercise-induced fatigue (Zhang and Huang, 2013; Zhao and Li, 2017; Ma, 2019) and chronic fatigue syndromes (CFS) (Xiao et al., 2014; Lin and Jiang, 2017). Moxibustion has become a widely recognized alternative treatment for exercise-induced fatigue in contemporary clinical practice (Xu et al., 2014). Previous studies showed that moxibustion alleviated exercise-induced fatigue by protecting against oxidative damage in skeletal muscle (Xu et al., 2014), alleviating cardiac injury (Zhang et al., 2015), and reducing the serum pro-inflammatory cytokines in rats (Lu et al., 2012). However, it remains unclear whether moxibustion can alleviate the inflammatory response in the brain regions of exhaustive exercise-induced fatigue rats.

The hippocampus is one of the brain regions that is vulnerable to exhaustive stress and inflammation. Neuroimaging studies have suggested that the neuroinflammation is present in widespread brain areas of CFS patients (Nakatomi et al., 2014). In this study, we investigated the effects of moxibustion on pro-inflammatory cytokines (IL-1β, IL-6, and TNF-α) response in the hippocampus of chronic exhaustive swimming -induced fatigue rats. We first investigated the effect of moxibustion on behavioral activities. Cytokines mRNA expression of IL-1β, IL-6, and TNF-α in the hippocampus was detected by RT-PCR, and these cytokines content was measured by ELISA. Our results suggest that moxibustion could attenuate exercise-induced fatigue and inhibits inflammation in the hippocampus in fatigue rats exposed to chronic exhaustive exercise.

## Materials and Methods

### Animals

Male sprague-dawley (SD) rats, 6–8 weeks old, weighing 180 to 200 g (Beijing Vital River Laboratories, China) were used in this study. The experimental animal license number is SCXK (Beijing) 2016-0011. The rats were housed in an air-conditioned room with a 12 h light/dark cycle with free access to food and water. Our study conformed to the guidelines of the China legislations on the ethical use and care of laboratory animals and was approved by the Animal Care and Use Committee at Beijing University of Chinese Medicine. Efforts were made to minimize the number of animals used and their suffering.

### Experimental Groups

The rats were randomly assigned to three groups as follows: the sedentary group (Sed, *n* = 8) was not exercised and was handled every day to minimize handling stress; the exercise-induced fatigue group (Model, *n* = 8) was subjected to the exhaustive swimming for 3 weeks; the exhaustive trained moxibustion group (Moxa, *n* = 8) was given the same exhaustive swimming as the Model group, and received moxibustion treatment once every other day for 11 times.

### Exercise-Induced Fatigue Induced by Exhaustive Exercise Procedure

The chronic exhaustive swimming procedure was modified from the methods previously described (Ikeuchi et al., 2006). The rats were adapted to the 20 min swimming (at 30 ± 2°C) once a day for 3 continuous days before the actual exhaustive swimming procedure. The rats were placed in a cylindrical-like water tank (52 cm in diameter, 70 cm in height) in which rats could not support themselves by touching the bottom with their feet. Then the rats were exposed to daily exhaustive swimming for 3 weeks, and a lead fish sinker weighing approximately 5% of their body weight was attached to the tail. The total exhaustive swimming time of rats was calculated from the moment they were dropped into water until they were completely exhausted as assessed by failing to rise to the surface to breathe within 10 continuous seconds.

### Moxibustion Treatment

Moxibustion was administrated as described previously (Lu et al., 2012). Briefly, rats were maintained within a cloth bag with hindlimbs out. Acupoint Zusanli (ST36) was selected. Rats ST36 is located at 5 mm below the fibular head, posterior lateral to the knee joint. The skin surface of the acupoint was made hairless to enable the pedestal to be stuck on. The moxa cone (7 mm diameter × 10mm length, Beijing Zhongyan Taihe Medical Instrument Co., Ltd, China) was applied to the skin surface of the acupoint. The moxibustion treatment was performed for 3 consecutive runs (1 run for approximately 3 min) on the bilateral ST36 of rats after the exhaustive swimming, and once every other day during the 3-week period.

### Body Weight

The rats were weighed before the onset of the swimming procedure, and at the end of the 3-week exhaustive period.

### Exhaustive Swimming Time

The swimming time to exhaustion was observed before the exhaustive regimen, and at the end of the 3-week stress period. The total exhaustive swimming time of rats was calculated from the moment they were dropped into water until they were completely exhausted as assessed by failing to rise to the surface to breathe within 10 continuous seconds.

### Tail Suspension Test

The tail suspension test used to measure the total duration of immobility has been traditionally used to test depression-like behaviors (Crowley et al., 2006). Rats were suspended by the tail, using adhesive Scotch tape to a hook. The total duration of immobility was calculated during a 5-min test. The test was observed at the end of the 3-week exhaustive stress period.

### Open Field Test

The open field test is used to measure locomotor activity in rodents. A single rat was gently placed in the center of the floor of the locomotor activity apparatus (Ugo Basile, Italy) and allowed to freely explore the arena for 5 min. The crossing distance and the rearing distance were counted automatically by the apparatus. After each test, the field was cleaned thoroughly with a dilute alcohol solution.

### Sample Collection

After the behavioral tests, the rats were sacrificed under deep anesthesia. The brains were quickly removed, and the hippocampus was isolated on ice plate. All these samples were stored at −80°C for further analysis.

### Total RNA Isolation and Real Time RT-PCR

The mRNA expression of IL-1β, IL-6, and TNF-α in the brain was measured by RT-PCR as previously described (Guo et al., 2009). Briefly, the total RNA from brain was obtained by TRIzol method. RNA was reverse transcribed to cDNA using the Taqman^®^ Reverse Transcription Reagents (Applied Biosystems, United States). Real-time quantitative PCR analyses for IL-1β, IL-6, and TNF-α were performed in 96-well plates using the ABI PRISM 7700 Sequence Detection System instrument and software (PE Applied Biosystems). PCR were performed with the SYBR Green PCR Master Mix (Applied Biosystems) according to the manufacturer’s protocol, using the following oligonucleotide primers: IL-1β: forward, 5′-GAGTCTGCACAGTTCCCCAA-3′; reverse, TGTCCCGACCATTGCTGTTT-3′(88bp); IL-6: forward, 5′-CACTTCACAAGTCGGAGGCT-3′; reverse, 5′- TCTGACA GTGCATCATCGCT-3′(114bp); TNF-α: forward, 5′- CGTC AGCCGATTTGCCATTT-3′; reverse, 5′-CTCCCTCAGGGG TGTCCTTA-3′(89bp); β-actin: forward, 5′-ACGTTGACATCC GTAAAGAC-3′; reverse, 5′-GGACTCATCGTACTCCTG CT-3′(81bp).

The basic protocol for real-time PCR was an initial incubation at 95°C for 5 min, followed by 45 cycles of 95°C for 30 s, 56°C for 30 s, 72°C for 1 min and finally cooling to 40°C. All samples were run in triplicate and the relative expression values were normalized with β-actin value. Plasmids containing cDNA was used as standard in quantifying the PCR results. The interest cDNA was amplified by RT-PCR using the same premiers as for real-time RT-PCR. The PCR products were cloned into pGEM-T easy vector (Invitrogen) and confirmed by sequencing. The purified recombinant plasmid DNA was quantified by UV spectrophotometer and then serially diluted in double-distilled water as standard for numerical quantification. The PCR products were sequenced to verify the analytical specificity. Melting curve was analyzed after PCR amplification.

### Enzyme-Linked Immunosorbent Assay

The tissue was dissociated using an ultrasonic cell disrupter and lysed in cold lysis buffer containing 10 mM Tris-HCl, pH 8.0, 240 mM NaCl, 5 mM EDTA, 1 mM dithiothreitol, 0.1 mM phenylmethylsulfonyl fluoride, 1% Triton X-100, 1 mM sodium vanadate and 1 g/ml of leupeptin, pepstatin, aprotinin. Tissue lysates were incubated at 4°C for 20 min. The sample was centrifuged at 12,000 rpm for 10 min at 4°C, then the supernatant was collected, and the protein content was determined by BCA protein assay reagents (Pierce, Rockford, IL) following the manufacturer’s protocol. The concentrations of IL-1β, IL-6, and TNF-α in the hippocampus, prefrontal cortex and serum were measured using commercially available Enzyme-Linked Immunosorbent Assay (ELISA) kits (Multisciences Biological Technology, China) according to the manufacturer’s instructions.

### Statistical Analysis

Data are expressed as mean ± SEM. Differences among groups were examined using a one-way analysis variance (ANOVA) followed by Newman-Keuls test. The independent-samples t test was used for comparing the swimming time between the Model group and the Moxa group. *P* < 0.05 was considered statistically significant.

## Results

### Effects of Moxibustion Treatment on the Body Weight

**FIGURE 1:**
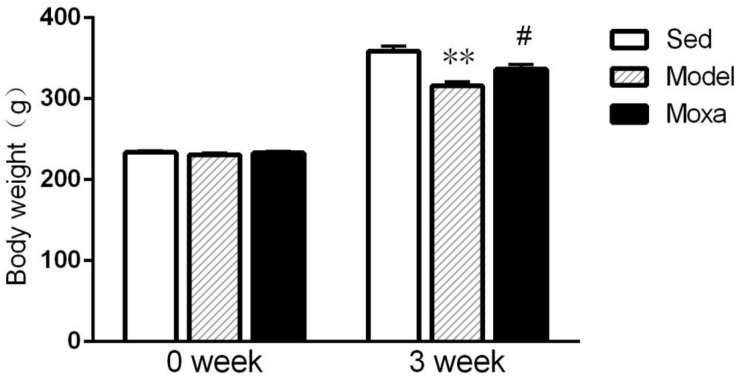
Body weight in the following groups (*N* = 8 per group): Sedentary (Sed), Model, Moxibustion (Moxa). Data are means ± SEM. ^∗∗^*P* < 0.01 as compared with the Sed group, ^#^*P* < 0.05 as compared with the Model group.

As illustrated in Figure 1, before the chronic exhaustive swimming period, rats from different groups showed no significant difference in body weight (*P* > 0.05). There was significant difference among groups following 3 weeks of chronic exhaustive swimming. The Model rats exhibited a reduction of body weight compared with Sedentary control after 3 weeks of exhaustive swimming (315.875 ± 5.22 g vs. 358.5 ± 6.66 g; *P* < 0.01). Moxibustion treatment significantly increased the body weight of rats compared with model rats (336.375 ± 5.65 g vs. 315.875 ± 5.22 g; *P* < 0.05).

### Effects of Moxibustion Treatment on the Exhaustive Swimming Time

**FIGURE 2:**
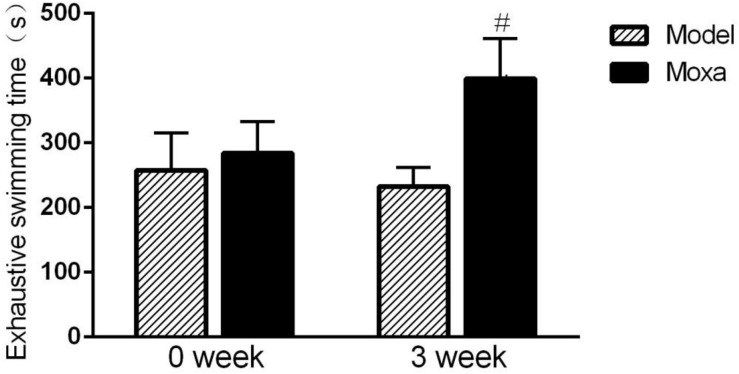
The chronic exhaustive swimming procedure lasted 3 weeks. The behavioral test was performed on week 0 and week 3 (day 1 and day 21, respectively). Exhaustive swimming in the following groups (*N* = 8 per group): Model, Moxibustion (Moxa). Data are means ± SEM. ^#^*P* < 0.05 as compared with the Model group.

As shown in Figure 2, there was no difference in exhaustive swimming time between model group and Moxa group on week 0. The exhaustive swimming time was significantly decreased in Model group compared with Moxa group after 3 weeks of chronic exhaustive swimming (232.25 ± 30.23 s vs. 399.13 ± 62.39 s; *P* < 0.05).

### Effects of Moxibustion Treatment on the Tail Suspension Immobility Time

**FIGURE 3:**
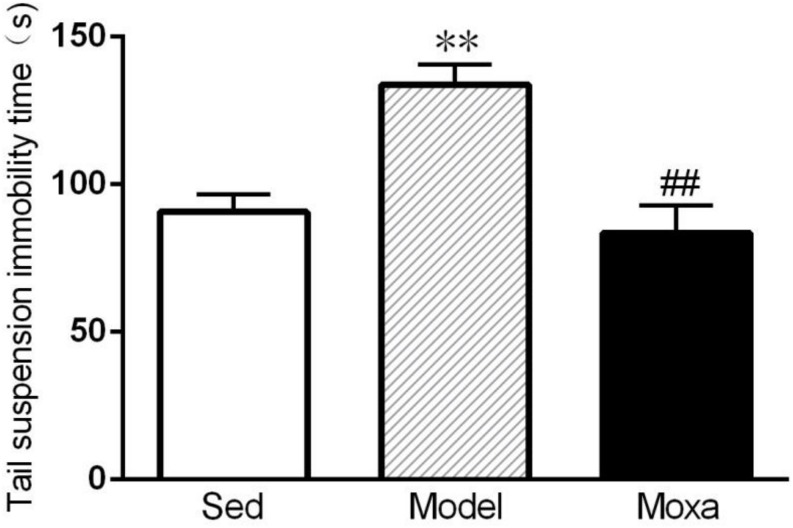
Tail suspension immobility time in the following groups (*N* = 8 per group): Sedentary (Sed), Model, Moxibustion (Moxa). Data are means ± SEM. ^∗∗^*P* < 0.01 as compared with the Sed group, ^##^*P* < 0.01 as compared with the Model group.

As shown in Figure 3, the tail suspension immobility time was significantly increased in Model group compared with Sedentary control (133.17 ± 13.20 s vs.96.5 ± 9.96 s; *P* < 0.01). Moxibustion treatment significantly decreased the tail suspension immobility time of rats compared with Model rats (79.5 ± 11.42 s vs. 133.17 ± 13.20 s; *P* < 0.01).

### Effects of Moxibustion Treatment on the Locomotor Activity in Open Field Test

**FIGURE 4:**
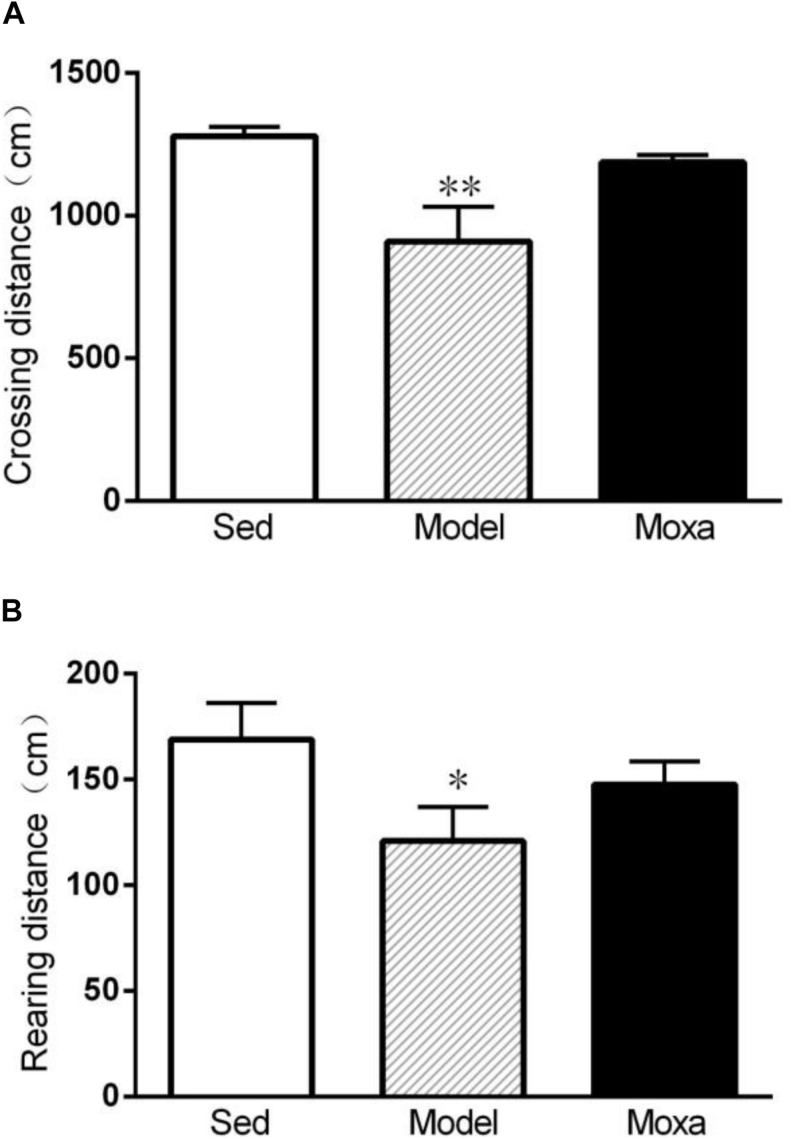
The locomotor activity of open field test in the following groups (*N* = 8 per group): Sedentary (Sed), Model, Moxibustion (Moxa). **(A)** Crossing distance. **(B)** Rearing distance. Data are means ± SEM. ^∗∗^*P* < 0.01 or ^∗^*P* < 0.05 as compared with the Sed group.

A significant reduce was observed in Model group compared with Sedentary control both in crossing distance (908.875 ± 113.78 cm vs.1278.5 ± 29.69 cm; *P* < 0.01) and rearing distance (120.875 ± 15.06 cm vs.168.875 ± 16.13 cm; *P* < 0.05). However, moxibustion treatment had no effect on crossing distance and rearing distance (*P* > 0.05). As shown in Figure 4.

### Effects of Moxibustion Treatment on Cytokine mRNA Expression in the Hippocampus

**FIGURE 5:**
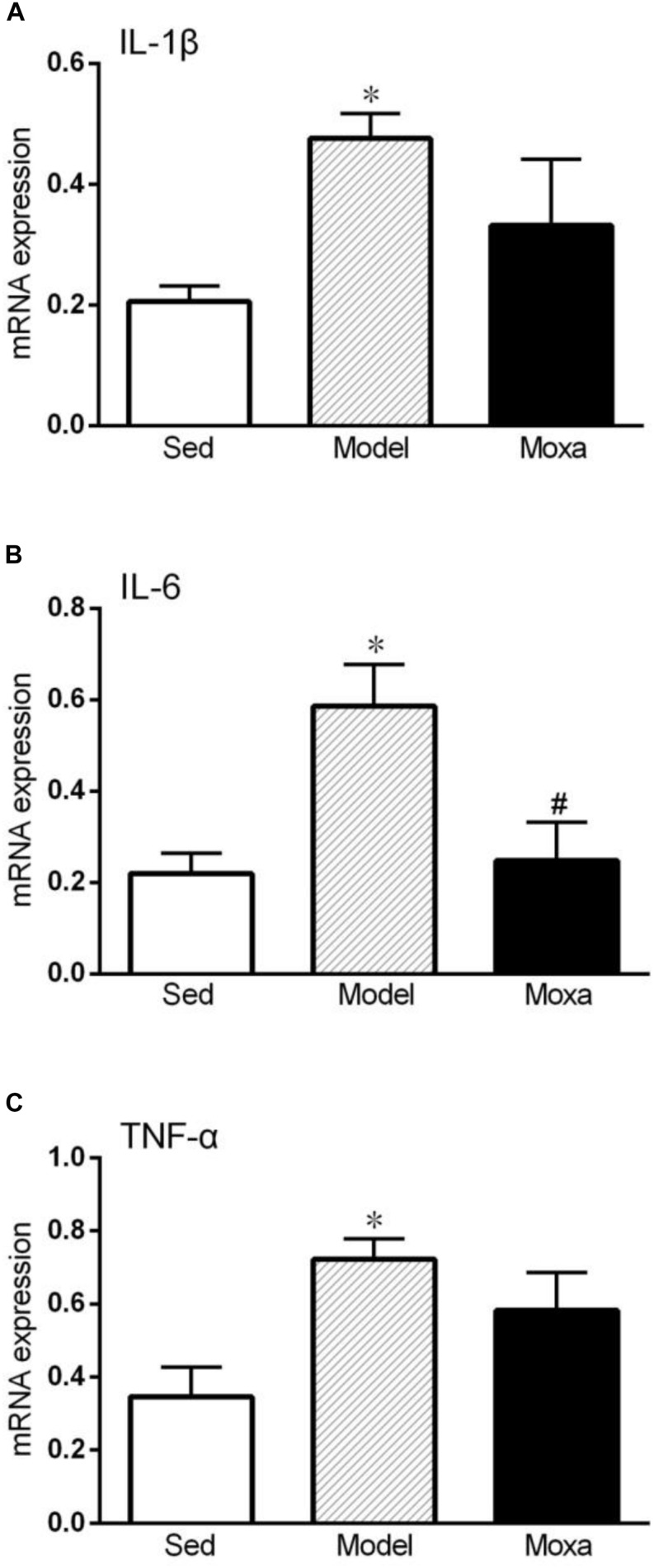
Hippocampus cytokines gene expressions measured by RT-PCR in the following groups: Sedentary (Sed, *N* = 5), Model (*N* = 5), Moxibustion (Moxa, *N* = 4). **(A)** Hippocampus gene expression level of IL-1β. **(B)** Hippocampus gene expression levels of IL-6. **(C)** Hippocampus gene expression levels of TNF-α. Data are means ± SEM. ^∗^*P* < 0.05 as compared with the Sed group, ^#^*P* < 0.05 as compared with the Model group.

As shown in Figure 5, the IL-1β mRNA expression in the hippocampus of model was significantly higher than that of Sedentary control (0.47 ± 0.04 vs. 0.21 ± 0.03; *P* < 0.05). The IL-6 mRNA expression in the hippocampus of model was significantly increased compared with Sedentary control (0.59 ± 0.09 vs. 0.22 ± 0.04; *P* < 0.05). The TNF-α mRNA expression in the hippocampus of model (0.72 ± 0.05 vs. 0.34 ± 0.08; *P* < 0.05) was also significantly increased compared with Sedentary control. Moxibustion treatment significantly reduced the mRNA expression of IL-6 (*P* < 0.05), while it did not significantly reduce the mRNA expression of IL-1β and TNF-α (*P* > 0.05) in the hippocampus.

### Effects of Moxibustion Treatment on Cytokines Level in the Hippocampus

**FIGURE 6:**
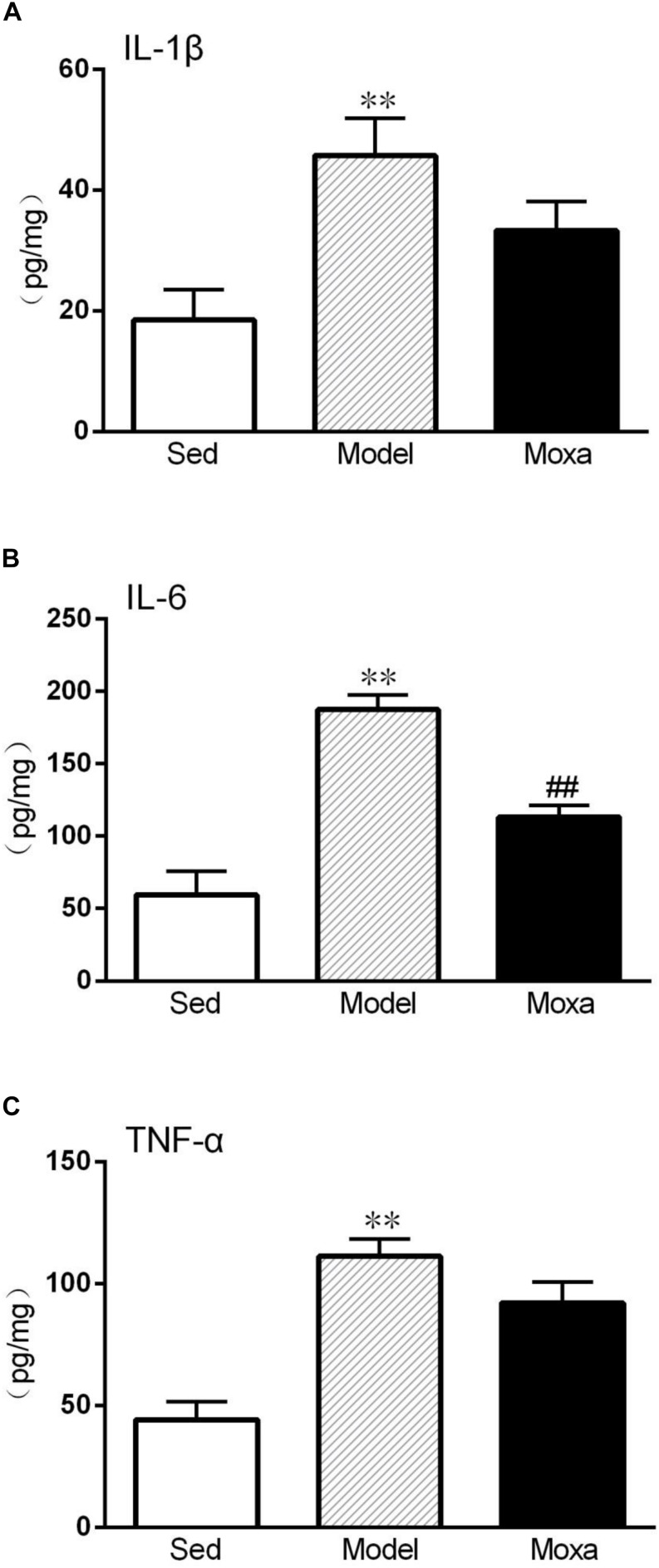
Hippocampus cytokines levels measured by ELISA in the following groups (*N* = 8 per group): Sedentary (Sed), Model, Moxibustion (Moxa). **(A)** Hippocampus level of IL-1β. **(B)** Hippocampus level of IL-6. **(C)** Hippocampus level of TNF-α. Data are means ± SEM. ^∗∗^*P* < 0.01 as compared with the Sed group, ^##^*P* < 0.01, as compared with the Model group.

We further detected the cytokines protein level in the hippocampus. The IL-1β level in the hippocampus of model (45.78 ± 5.77 pg/mg vs. 18.46 ± 4.72 pg/mg; *P* < 0.01) was significantly higher than that of Sedentary control. The IL-6 level in the hippocampus of model (187.35 ± 9.63 pg/mg vs. 59.49 ± 15.11 pg/mg; *P* < 0.01) was significantly increased compared with Sedentary control. The TNF-α levels in the hippocampus of model (111.30 ± 6.69 pg/mg vs. 44.15 ± 6.90 pg/mg; *P* < 0.01) were also significantly increased compared with Sedentary control. Moxibustion treatment significantly reduced the contents of IL-6 (*P* < 0.01) but did not significantly reduced the contents of IL-1β and TNF-α (*P* > 0.05) in the hippocampus, as shown in Figure 6.

## Discussion

The aim of this study was to investigate potential anti-inflammatory effect of moxibustion in treating exercise-induced fatigue. Our results showed that the pro-inflammatory cytokines (IL-1β, IL-6, TNF-α) levels were significantly increased in the hippocampus of exercise-induced fatigue rat. Moxibustion treatment exhibits anti-fatigue effect in exhaustive swimming rat model of exercise-induced fatigue. Furthermore, we found that this effect might be mediated by inhibition of pro-inflammatory cytokine IL-6 levels in the hippocampus.

It has been well recognized that high-load training with insufficient recovery enhances the production and release of IL-1β, IL-6, and TNF-α both in serum and central nervous system (Castell et al., 2019). The proinflammatory cytokines produced in the brain could induce sickness behavior, such as fatigue and depressive-like behavior. In the study, we found that swimming time to exhaustion was reduced by chronic weight-loaded exhaustive swimming. Chronic exhaustive exercise also reduced body weight and locomotor activity in open field test, while increased immobility time of tail suspension test. A reduction of swimming time reflects the impairment of exercise performance and endurance capacity, which is one of the major symptoms of exercise-induced fatigue. The exhaustive stress-induced decrease in body weight is an accompanying symptom of exercise-induced fatigue rat. An increase of immobility time of tail suspension test may indicate the depressant-like activity (Cryan et al., 2005). A reduction of the locomotor activity may indicate a lower desire to explore. We found that moxibustion treatment improved exhaustive swimming time and the body weight, as well as reducing immobility time. However, it did not significantly affect the locomotor activity. Moxibustion has been shown to alleviate depression-like behavior in chronic stress rat model of depression (Ding et al., 2016). These results demonstrated that moxibustion could have the effect of increasing exercise performance and alleviating the depressant-like activity in exercise-induced fatigue rat model.

Increasing evidence has recently indicated that chronic inflammatory response plays a key role in the pathophysiology of exercise-induced fatigue (Vargas and Marino, 2014). The practice of regular, non-exhaustive physical activity improves health conditions and have beneficial effects on immunity (Dantas et al., 2019). However, excessive training with insufficient recovery causes musculoskeletal trauma with increased production of pro-inflammatory cytokines such as IL-1β, IL-6, and TNF-α, which contributes to symptoms related to performance decrement and exercise-induced fatigue (Smith, 2000, 2004; Carmichael et al., 2010). The pro-inflammatory cytokines play important roles in modulating immune and inflammatory responses to exercise. Studies have shown that several pro-inflammatory cytokines in the plasma were increased after strenuous exercise (Giraldo et al., 2008; Main et al., 2009). Prolonged and intensive running increased IL-6 and IL-8 levels in the plasma and muscle of runners (Nieman et al., 2015). In parallel with human studies, reports have shown an increase in pro-inflammatory cytokines in peripheral tissues and brain in over-trained rats. For example, the serum IL-1β was increased by chronic running wheel exercise in rats (Rowsey et al., 2009). Excessive eccentric running resulted in an increase in the serum levels of IL-1β, IL-6, and TNF-α in mice (Pereira et al., 2015). Over-expression of IL-1β has also been observed in the mice brain regions after an eccentric exercise, and the IL-1β levels was significantly correlated with post-exercise delayed recovery and decreased physical performance (Carmichael et al., 2005, 2010). In addition, wheel running activity in mice was significantly decreased by intracerebroventricular injection of IL-1β, while the administration of IL-1 receptor antagonist could reverse the performance deficit (Carmichael et al., 2006). Consistent with previous findings (Pereira et al., 2015), our study showed that the mRNA and protein expression of IL-1β, IL-6 and TNF-α in the hippocampus were significantly increased in exhaustive swimming rats. It has been well accepted that the increase in pro-inflammatory cytokines in the brain has been linked to sickness behaviors including fatigue. It has been reported that CFS is accompanied by high levels of peripheral inflammatory cytokines and persistent mental and physical fatigue (Morris et al., 2015). Chronic fatigue in athletes suffering from overtraining may also result from peripheral pro-inflammatory cytokines and neuroinflammation in the brain (Smith, 2000). It suggested that exercise-induced fatigue might result from both peripheral and central inflammatory mechanism.

It has been well documented that moxibustion could modulate immune function in various types of illness. For example, moxibustion could attenuate intestinal inflammation and promote the repair of colon mucosal injury in a rat model of Crohn’s disease (Zhao et al., 2019). Moxibustion could regulate serum TNF-α level and reduce foot swelling in rats with rheumatoid arthritis (Wu et al., 2018). In addition, it has been reported that moxibustion could regulate the Th1/Th2 cytokine imbalance of athletes during the course of heavy load training (Gao et al., 2011). Previous study has reported that moxibustion treatment could regulate pro-inflammatory cytokines contents in the serum of exhaustive exercise rats (Lu et al., 2012). However, the effect of moxibustion on central pro-inflammatory cytokines is not clear.

We found that moxibustion treatment significantly decreased IL-6 mRNA and protein levels in the hippocampus of exhaustive exercise-induced fatigue rats. During exercise, IL-6 is usually the first cytokine released from the contracting skeletal muscle cells into the blood. It has been recognized that the impact of muscle-derived IL-6 plays a major role in the development of central fatigue (Gleeson, 2000). However, the increased IL-1β and TNF-α level in the hippocampus following chronic exhaustive exercise was not significantly reduced by moxibustion treatment. A previous study has reported that perivascular and meningeal macrophages is the major producer of brain IL-1β during exercise (Carmichael et al., 2010). It has been shown that TNF-α gene expression in circulating mononuclear cells was increased after heavy exercise training (Moldoveanu et al., 2000). It seems that moxibustion did not affect macrophages or mononuclear cells significantly. These results indicated that moxibustion treatment could inhibit over-expression of pro-inflammatory cytokine IL-6 levels in the brain and alleviate inflammation in chronic exhaustive exercise rat.

## Conclusion

In summary, our data suggested that moxibustion may exhibit the anti-fatigue effect on chronic exhaustive exercise-induced fatigue rat, which might be attributed to inhibiting pro-inflammatory cytokine IL-6 levels and reducing inflammation in the brain region.

## Data Availability

The datasets generated for this study are available on request to the corresponding author.

## Ethics Statement

The animal study was reviewed and approved by the Animal Care and Use Committee at Beijing University of Chinese Medicine.

## Author Contributions

TL and LS: collection and assembly of behavioral tests, and writing of the manuscript. DG and RP: ELISA and RT-PCR detection. SB: moxibustion treatment. JL: study design, data analysis, and manuscript writing. YC: study design and data analysis.

## Conflict of Interest Statement

The authors declare that the research was conducted in the absence of any commercial or financial relationships that could be construed as a potential conflict of interest.
